# Oncofetal Protein CRIPTO Is Involved in Wound Healing and Fibrogenesis in the Regenerating Liver and Is Associated with the Initial Stages of Cardiac Fibrosis

**DOI:** 10.3390/cells10123325

**Published:** 2021-11-26

**Authors:** Sofia Karkampouna, Danny van der Helm, Mario Scarpa, Bart van Hoek, Hein W. Verspaget, Marie-Jose Goumans, Minneke J. Coenraad, Boudewijn P.T. Kruithof, Marianna Kruithof-de Julio

**Affiliations:** 1 Department for Biomedical Research, Urology Research Laboratory, Bern University, 3008 Bern, Switzerland; sofia.karkampouna@dbmr.unibe.ch (S.K.); mario.scarpa@dbmr.unibe.ch (M.S.); 2 Department of Gastroenterology and Hepatology, Leiden University Medical Center, 2333 ZA Leiden, The Netherlands; d.van_der_helm@lumc.nl (D.v.d.H.); B.van_Hoek@lumc.nl (B.v.H.); h.w.verspaget@lumc.nl (H.W.V.); m.j.coenraad@lumc.nl (M.J.C.); 3 Department of Cell and Chemical Biology, Leiden University Medical Center, 2333 ZC Leiden, The Netherlands; M.J.T.H.Goumans@lumc.nl (M.-J.G.); b.p.t.kruithof@lumc.nl (B.P.T.K.); 4 Department of Cardiology, Leiden University Medical Center, 2333 ZA Leiden, The Netherlands; 5 Department of Urology, Inselspital, Bern University Hospital, 3010 Bern, Switzerland; 6 Translational Organoid Resource Core, Department for BioMedical Research, Bern University, 3008 Bern, Switzerland; 7 Bern Center for Precision Medicine, Inselspital, University Hospital of Bern, 3010 Bern, Switzerland

**Keywords:** CRIPTO, fibrosis, tissue regeneration

## Abstract

Oncofetal protein, CRIPTO, is silenced during homeostatic postnatal life and often re-expressed in different neoplastic processes, such as hepatocellular carcinoma. Given the reactivation of CRIPTO in pathological conditions reported in various adult tissues, the aim of this study was to explore whether CRIPTO is expressed during liver fibrogenesis and whether this is related to the disease severity and pathogenesis of fibrogenesis. Furthermore, we aimed to identify the impact of CRIPTO expression on fibrogenesis in organs with high versus low regenerative capacity, represented by murine liver fibrogenesis and adult murine heart fibrogenesis. Circulating CRIPTO levels were measured in plasma samples of patients with cirrhosis registered at the waitlist for liver transplantation (LT) and 1 year after LT. The expression of CRIPTO and fibrotic markers (αSMA, collagen type I) was determined in human liver tissues of patients with cirrhosis (on a basis of viral hepatitis or alcoholic disease), in cardiac tissue samples of patients with end-stage heart failure, and in mice with experimental liver and heart fibrosis using immuno-histochemical stainings and qPCR. Mouse models with experimental chronic liver fibrosis, induced with multiple shots of carbon tetrachloride (CCl_4_) and acute liver fibrosis (one shot of CCl_4_), were evaluated for CRIPTO expression and fibrotic markers. CRIPTO was overexpressed in vivo (Adenoviral delivery) or functionally sequestered by ALK4Fc ligand trap in the acute liver fibrosis mouse model. Murine heart tissues were evaluated for CRIPTO and fibrotic markers in three models of heart injury following myocardial infarction, pressure overload, and ex vivo induced fibrosis. Patients with end-stage liver cirrhosis showed elevated CRIPTO levels in plasma, which decreased 1 year after LT. Cripto expression was observed in fibrotic tissues of patients with end-stage liver cirrhosis and in patients with heart failure. The expression of CRIPTO in the liver was found specifically in the hepatocytes and was positively correlated with the Model for End-stage Liver Disease (MELD) score for end-stage liver disease. CRIPTO expression in the samples of cardiac fibrosis was limited and mostly observed in the interstitial cells. In the chronic and acute mouse models of liver fibrosis, CRIPTO-positive cells were observed in damaged liver areas around the central vein, which preceded the expression of αSMA-positive stellate cells, i.e., mediators of fibrosis. In the chronic mouse models, the fibrosis and CRIPTO expression were still present after 11 weeks, whereas in the acute model the liver regenerated and the fibrosis and CRIPTO expression resolved. In vivo overexpression of CRIPTO in this model led to an increase in fibrotic markers, while blockage of CRIPTO secreted function inhibited the extent of fibrotic areas and marker expression (αSMA, Collagen type I and III) and induced higher proliferation of residual healthy hepatocytes. CRIPTO expression was also upregulated in several mouse models of cardiac fibrosis. During myocardial infarction CRIPTO is upregulated initially in cardiac interstitial cells, followed by expression in αSMA-positive myofibroblasts throughout the infarct area. After the scar formation, CRIPTO expression decreased concomitantly with the αSMA expression. Temporal expression of CRIPTO in αSMA-positive myofibroblasts was also observed surrounding the coronary arteries in the pressure overload model of cardiac fibrosis. Furthermore, CRIPTO expression was upregulated in interstitial myofibroblasts in hearts cultured in an ex vivo model for cardiac fibrosis. Our results are indicative for a functional role of CRIPTO in the induction of fibrogenesis as well as a potential target in the antifibrotic treatments and stimulation of tissue regeneration.

## 1. Introduction

Fibrotic diseases are responsible for 45% of deaths in the developed world [1]; therefore, understanding the regulatory pathways involved in organ fibrosis is a necessary step. Fibrosis is the increased secretion and deposition of extracellular matrix (ECM) that will lead to perturbation of the normal tissue architecture and eventually to organ dysfunction [2,3]. In some organs/tissues the fibrotic tissue can be (partly) resolved by regenerative processes, as seen in liver fibrosis. In other organs, the fibrosis is permanent due to the inability of the organ to regenerate, as seen in the heart. Whereas fibrosis can occur in many different organs/tissues and involves the interplay of many different cell types and factors, the fibrotic process in the different organs/tissues shares many similarities including their triggers, the activation of myofibroblasts (MFBs), inflammation cascade, and the activation of the Transforming Growth Factor-beta (TGF-β) pathway [1].

ECM-producing MFBs are the key mediators of tissue remodelling and are characterized by αSMA expression [4,5,6]. The source of the MFBs is organ dependent [7]. For example, in the liver they derive from the hepatic stellate cells, i.e., pericytes found in the space of Disse, whereas in the heart they derive from the existing interstitial fibroblasts [8,9]. The activation of the MFBs is often the result of chronic exposure to damaging factors.

In the liver, the stellate cells are thought to be activated by damaged and apoptotic hepatocytes upon chronic exposure to damaging factors such as alcohol and viral hepatitis B or C (HBV, HCV) [10]. Approximately 10%–30% of chronic liver diseases progress to cirrhosis, which is associated with high mortality and health care burden [11]. The excessive collagen deposition disrupts liver architecture, leading to hepatocellular dysfunction [2,12,13,14], causing cirrhosis and eventually increasing the risk of hepatocellular carcinoma (HCC) development.

During myocardial infarction (MI), cardiomyocyte death leads to the generation of many inflammatory and profibrotic factors that can activate the interstitial fibroblasts. The MFBs subsequently form a fibrous scar, which is critical to protect the heart from rupture. Hypertension or aortic stenosis, on the other hand, causes increased ventricular pressure, leading to the activation of MFBs and subsequent formation of progressive interstitial and perivascular fibrosis [15]. The fibrosis in the heart triggers myocardial stiffness, resulting in ventricular dysfunction, which could ultimately result in heart failure [3].

Hepatic fibrosis, advanced cirrhosis, and cardiac fibrosis are major health problems, with a lack of effective antifibrotic treatment options [16,17,18,19]. For liver fibrosis, withdrawal of the injuring stimulus is the only current treatment, which in some cases leads to the resolution of fibrogenesis [20]. For end-stage liver cirrhosis, liver transplantation (LT) is still the only curative treatment option (feasibility depends on patient condition and donor availability), even if it is still a major surgical intervention with substantial risk of complications and risk of disease recurrence [21,22]. For cardiac fibrosis, current therapies aim to promote more functional scar formation and to improve heart function [23]; however, effective therapies directly targeting the process of fibrosis do not exist. Therefore, therapies directly targeting fibrosis are needed. Better understanding of the pathological mechanisms underlying fibrosis could lead to the identification of new biomarkers to monitor the disease and may also lead to new targets for the development of alternative treatment strategies.

CRIPTO (Teratocarcinoma-Derived Growth Factor-1; TDGF-1) is a GPI-anchored signaling protein, a member of the epidermal growth factor-Cripto/frl/cryptic (EGF-CFC) family, with diverse functions in embryogenesis and as regulator of stemness [24,25]. CRIPTO is silenced postnatally and often re-expressed in neoplasms of breast, lung, prostate, ovarian, bladder, colon, skin, and brain, where it is thought to be involved in cancer progression and metastasis [26,27,28,29,30,31,32,33,34,35]. Recently, we observed CRIPTO expression in the majority of cirrhotic liver tissues [36], which might indicate that reactivation of CRIPTO in adult tissues that is associated with pathological conditions such as inflammation, fibrosis, and (pre)malignant state. Therefore, we assessed in the present study whether CRIPTO is expressed during fibrosis and whether this is related to the disease severity. To determine whether CRIPTO expression in fibrosis is liver-specific or rather a general feature of fibrosis, we expanded our study to heart fibrosis.

CRIPTO expression was evaluated in liver cirrhosis specimens and in circulating blood levels of patients with cirrhosis prior to and after removal of the fibrogenic liver by LT. The observed correlation of CRIPTO expression with disease stage suggests a functional role for CRIPTO in the fibrosis-cirrhosis-HCC cascade, rendering it as a potential and interesting marker for disease monitoring or even as a novel treatment target. CRIPTO expression was further evaluated in validated mouse models for acute and chronic liver fibrosis: chronic or single administration of hepatotoxin carbon tetrachloride (CCl_4_), respectively. In three models of heart injury following myocardial infarction, pressure overload, and ex vivo induced fibrosis, CRIPTO was found to be temporally upregulated. Our findings indicated that CRIPTO is an immediate wound-healing response gene, following tissue injury of different aetiologies and it is a master orchestrator of fibrogenesis in multiple tissues as determined in human cirrhosis and murine hepatic models, as well as in vivo and ex vivo cardiac fibrosis. Our data implicated CRIPTO as a potential marker for disease monitoring and a treatment target of organ fibrosis.

## 2. Materials and Methods

### 2.1. Patients and Controls

Plasma CRIPTO levels were measured in paired pre- and 1 year post-LT plasma samples from consecutive patients with end-stage liver disease due to alcoholic cirrhosis (ALD, *N* = 25) or viral hepatitis-induced cirrhosis (*N* = 20) who had an available plasma sample just before transplantation. CRIPTO levels measured in plasma from healthy volunteers (*N* = 16) served as control. Exclusion criteria for this study were the presence of HCC, a combined etiology of cirrhosis, death or re-LT within 1 year after LT, and the development of serious adverse events after LT, such as Tacrolimus-induced renal insufficiency (Table S1: patient characteristics). For qPCR and (immuno)-histochemical analysis, control tissue (*N* = 5) and alcohol- or viral hepatitis-induced fibrotic/cirrhotic liver tissues (*N* = 19) were obtained from the tissue collections of the LUMC Liver Diseases Biobank and Pathology department. These tissues were derived from different patients and, later on, these patients were included in the circulating plasma CRIPTO study. All liver tissues were obtained during LT or resection of HCC or colorectal cancer-derived liver metastasis.

Clinical data were extracted from electronic patient files, including laboratory assessments and clinical MELD (Model for End-Stage Liver Disease) scores, a scoring system for assessing liver function impairment in cirrhosis and risk of short-term mortality. Cardiac samples were resected from the left ventricle of patients with end-stage heart failure (kindly provided by Dr. Bax). All experiments with human specimens were approved by the ethical research committee of the Leiden University Medical Center (LUMC, protocol number: B15.006). Materials were used in compliance with the rules prescribed by the regulations of the LUMC Liver Diseases Biobank and with a signed, informed consent of the donors.

### 2.2. Mouse Models of Fibrosis

All animal experiments were performed in C57BL6 mice in compliance with the guidelines for animal care and approved by the LUMC Animal Care Committee. Mice received food and water *ad libitum* and were housed under 12-h day/night cycle. Liver fibrosis was induced in 6-week-old male mice, as described previously [37]. For a period of 11 weeks, mice received two intraperitoneal (i.p.) injections per week with carbon tetrachloride (CCl_4_) in mineral oil (Sigma-Aldrich Chemie BV, 319961 Zwijndrecht, The Netherlands). The first week, mice received two initiating higher dosages of CCl_4_, of 200 µg/kg. The following 10 weeks, a maintenance dose of 150 µg/kg body weight was given twice weekly. All CCl_4_ injections were diluted to an injection volume of 50 µL with mineral oil. At the end of 11th week, mice were sacrificed and livers collected and subsequently fixated with 4% paraformaldehyde (PFA) for paraffin embedding or stored in isobutyl for RNA isolation.

Acute liver injury was induced in 5–6-week-old male C57Bl6 mice weighing 20–25 g by i.p. injection of a single dose of 1 mL/kg body weight of carbon tetrachloride (CCl_4_) (Sigma, 319961, stock concentration 1.594 g/mL), mixed 1:1 with mineral oil (final concentration 0.797 g/kg body weight) or mineral oil alone as control. Mice were sacrificed after 3, 6, 24, 48, 72 h, and day 6 (*n* = 2–3 per time point). 

Sections of mouse hearts with induced MI or TAC were kindly provided by Dr. Smits. MI was induced in 10–12-week-old mice by ligation of the left anterior descending artery (LAD), as described previously [38]. Hearts were collected at 1, 3, 7, 14, and 28 days post-MI. Pressure overload of the left ventricle was induced by transverse aortic constriction (TAC), which was performed as described previously [39]. Hearts were isolated at 2, 6, and 8 weeks post-TAC. Ex vivo culturing of adult mouse hearts was performed as described [40,41] using the ligation of the aorta and induction of retrograde flow of 1000 µL/min for 7 days. Hearts were fixed overnight in 4% PFA/PBS at 4 °C.

### 2.3. In Vivo Administration of Adenovirus

Adenoviral constructs expressing β-galactosidase (lacZ) or Cripto coding sequence were prepared using the Gateway adenoviral expression vectors pAd/CMV/V5-lacZ or pAd/CMV/V5-DEST; mouse Cripto expression plasmid was a gift from from Dr. Peter Gray [42]. AdlacZ (control) or AdCripto (1 × 10^9^ viral particles/mouse) was injected intravenously via the tail vein to enhance delivery to the liver [43]. After 24 h, CCl_4_ was injected i.p. (day 0). Treatment groups were as follows: AdlacZ, CCl_4_+AdlacZ, AdCripto, CCl_4_+AdCripto. At day 1, day 2, and day 3 after CCl_4_ administration, mice were sacrificed and liver tissues were collected for histology preparation, RNA/protein isolation, and analysis.

### 2.4. Preparation of ALK4Fc

A purified ALK4Fc (specifically ALK4L75A-Fc) ligand trap was produced, as previously described [26,44], from 293T-conditioned media using sequential protein A and FLAG affinity chromatography.

### 2.5. Histological Examination of Fibrosis

Tissues fixed in PFA were dehydrated through a graded series of ethanol, cleared in xylene, embedded in paraffin, and sectioned at 4–6 μm. To evaluate the severity of fibrosis in human and mouse liver tissue, a Sirius-red staining was performed to visualize the amount of collagen deposition. Paraffin sections (4 µm) were hydrated and subsequently stained for 90 min with 1 g/L Sirius-red F3B in saturated picric acid (both Klinipath). Next, the sections were incubated for 10 min with 0.01 M HCL, dehydrated, and mounted with Entellan (Merck KGaA, Darmstadt, Germany). Fixed microscope settings were used to capture 5–8 representative images (10× magnifications) that were subsequently used to quantify the amount of staining with ImageJ software (ImageJ 1.47v, National Institutes of Health, USA). With fixed threshold settings, based on control tissues, positive pixels were measured and the respective percentage to the total image was calculated and defined as the positive area.

### 2.6. Immunohistochemistry, Immunofluorescence Imaging, and Quantification

Immunohistochemical stainings were performed in fibrotic and control human and mouse liver tissue. Briefly, paraffin tissue sections (4 µm) were hydrated and endogenous peroxidases were blocked with 0.3% H_2_O_2_/methanol (20 min). Antigen retrieval was performed by 10-min boiling in citrate buffer (0.1 M, pH 6.0). After cooling down, primary antibodies detecting mouse- and human-CRIPTO (both kindly provided by Dr. Gray Clayton Foundation Laboratories for Peptide Biology, The Salk Institute for Biological Studies, La Jolla, California, USA) and anti-αSMA antibodies (A2547, clone 1A_4_; Sigma-Aldrich, Buchs, Switzerland) were added and incubated overnight. Human-αSMA staining was visualized by 1-h incubation with a secondary goat anti-rabbit-HRP-conjugated antibody followed by a 10-min incubation with 3,3′-diaminobenzidine (DAB Fast Tablet, Sigma-Aldrich, Buchs, Switzerland). Nuclear counterstaining was performed with hematoxylin, after which the sections were dehydrated and mounted with Entellan.

**Figure 1:**
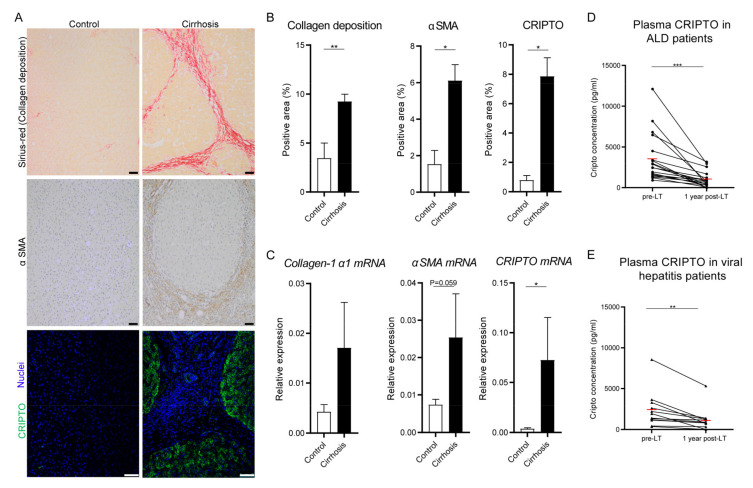
CRIPTO expression in patients with end-stage liver cirrhosis. Liver tissue samples of patients with ALD or virally induced liver cirrhosis (*N* = 19) and controls (*N* = 5) were randomly selected to evaluate CRIPTO expression. (**A**) Representative pictures of control and cirrhotic liver tissue stained for collagen deposition (Sirius-Red, scale bars 50 µm), αSMA (scale bars 50 µm), CRIPTO (green), and Nuclei stained with DAPI (blue) scale bars 75 µm). (**B**) Quantification of Sirius-red, αSMA, and CRIPTO staining (mean ± SEM). All samples were derived from the same experiment, and the staining and quantification were performed once per tissue sample. (**C**) The mRNA expression levels of *Collagen-1α1*, *αSMA*, and *CRIPTO* normalized to *β-actin* (mean ± SEM); * *p* ≤ 0.05 ** *p* ≤ 0.01. Tissues were derived from one experiment and measured with three technical repeats. *N* = 5–10 samples per group. (**D**,**E**) CRIPTO levels in plasma decreased after liver transplantation (LT) in different aetiological sub-cohorts. CRIPTO levels in pre- and post-LT paired plasma samples of patients suffering from (**D**) ALD- (*N* = 19) or (**E**) virally (*N* = 12) induced liver cirrhosis. Mean group levels are indicated by a red line; ** *p* ≤ 0.01 *** *p* ≤ 0.001. All samples were measured in duplicate.

**Figure 2:**
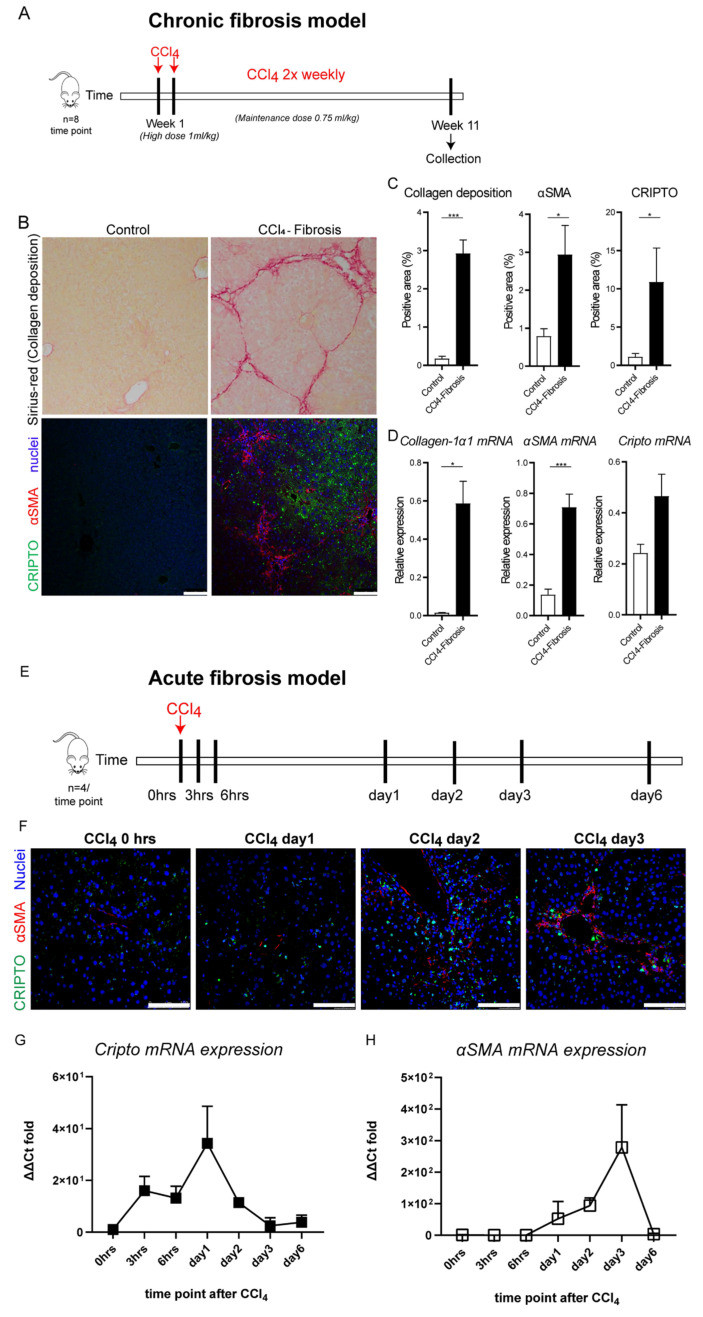
CRIPTO is upregulated in a CCl_4_ mouse model for chronic liver fibrosis and is reactivated in the early phase of acute CCl_4_ liver fibrosis. (**A**) Experimental setup. Mice received chronic administration (11 weeks) of CCl_4_ to induce liver fibrosis. (**B**) Representative pictures of healthy control and fibrogenic liver tissues stained for collagen deposition (Sirius-Red) and co-labeled for αSMA (red) and CRIPTO (green) (scale bars 75 μm). (**C**) Quantification of Sirius-Red, αSMA, and CRIPTO staining (*N* = 8 mice, mean ± SEM). All samples were derived from the same experiment, and the staining and quantification were performed once per tissue sample. (**D**) The mRNA expression levels of *collagen-1α1*, *αSMA*, and *Cripto* normalized to β-actin (*N* = 8 mice, mean ± SEM); * *p* ≤ 0.05 *** *p* ≤ 0.001. Tissues were derived from one experiment and were measured with three technical repeats; *n* = 5–10 samples per group. (**E**) Experimental setup. Mice received one shot of CCl_4_ (acute liver fibrosis model, 1 mg/kg) and liver tissues were collected at day 0; 0, 3, and 6 h, and day 1, day 2, day 3, and day 6 (*n* = 4 per time point). (**F**) Representative images of fibrotic liver tissues stained for αSMA (red) and CRIPTO (green) at day 0, day 1, day 2, and day 3. Nuclei were stained with TO-PRO-3. Scale bars, 100 μm. All samples were derived from the same experiment, and the staining and quantification were performed once per tissue sample. (**G**) The mRNA expression levels of *Cripto* and (**H**) *αSMA* normalized to *Gapdh* (Normalized ΔΔCt fold change to timepoint 0 h, mean ± SD). Tissues were derived from one animal experiment; PCR expression test was performed in two independent experiments, each with three technical replicates and *n* = 3 samples per group.

For immunofluorescence of the acute liver and heart models of fibrosis, tissues were fixed overnight in 4% PFA/PBS and paraffin sections were sectioned at 4 μm (liver tissues) or 6 μm (heart tissues). Antigen retrieval was performed in a pressure cooker (30 min for liver, 40 min for heart tissues) in Antigen Unmasking citrate-based solution (H-3300) (Vector laboratories, Burlingame, CA, USA), and sections were blocked in 1% BSA solution/0.01%Tween 20 in PBS. The following primary antibodies were added: CRIPTO and αSMA, anti-COLLAGEN type I (1:500, 1310-01, Southern Biotech, Birmingham, AL, USA) and anti-COLLAGEN type III (1:500, 1330-01, Southern Biotech, USA), and anti-Ki67 (1:200, clone SP6, GTX16667, GeneTex, LucernaChem, Luzern, Switzerland). The following day, sections were incubated with secondary antibodies labelled with Alexa Fluor 488, 555, or 647 (Invitrogen/Molecular Probes, Zurich, Switzerland; 1:250 in PBS/0.1% Tween 20). Tyramide Signal Amplification (PerkinElmer, Waltham, MA, USA) was used to amplify the CRIPTO signal followed by Alexa Fluor^®^ 488 streptavidin (Invitrogen/ Thermo Fisher Scientific, Basel, Switzerland) for visualization, as previously described [36]. Sections were counterstained with TO-PRO-3 (Invitrogen/Molecular Probes) or DAPI solution (Sigma-Aldrich, Buchs, Switzerland) for visualization of nuclei and mounted using Prolong G anti-fade mounting medium (Invitrogen/Molecular Probes). Representative pictures of the liver samples were captured using a confocal microscope (Leica Biosystems BV, Amsterdam, The Netherlands) and 40× 1.4 NA oil-immersion objective with fixed microscope and software settings. Subsequently, 5–10 representative pictures were captured and used for quantification. The amount of DAB or fluorescent staining in the representative pictures was quantified with ImageJ software. For the CRIPTO stainings in Figure 1 and Figure 2, the threshold was set, based on control tissues, and defined as a percentage of positive pixels compared to the total pixels within the hepatocyte regions, whereas regions such as the vessels, bile ducts, and the septa with ECM were excluded from the analysis. For the αSMA stainings, whole images were used to quantify the positive area. For the immunofluorescence quantification of CRIPTO, Collagen types I and III, and αSMA staining, the positive area was measured with fixed threshold settings and were represented as percentage. For the Ki67 quantification, the number of positive cells was normalized to the total number of nuclei (DAPI) on each section, and an average of three different fields of view areas was used for each liver tissue. All slides with cardiac samples were scanned with the Pannoramic 250 slide scanner (version1.23, 3DHISTECH Ltd.) and analyzed using Caseviewer (version 2.3, 3DHISTECH Ltd.).

### 2.7. RNA Isolation, cDNA Synthesis, and Quantitative Polymerase Chain Reaction (qPCR)

Mouse and human liver tissues were homogenised with UltraTurrax homogenizer (T25 basic, IKA) and TRIpure reagent (Roche). Subsequently, mRNA was isolated following TRIpure RNA isolation protocol. Promega standard protocol was used to synthesise cDNA from 1 µg RNA (Promega, Madison, WI, USA). *CRIPTO*, *COLLAGEN-1α1*, and *αSMA* expressions in human and mouse samples were measured by qPCR analysis. The qPCR reaction mixtures consisted of 5 μL of iQ SYBR Green supermix reagent (Bio-Rad Laboratories, Berkeley, CA, USA), 1 nM of primers, and 4 µL of cDNA. Results were normalized to β-actin for both mouse and human samples. The primer sequences are indicated in Table S2.

### 2.8. Cell Culture and Reporter Assay

Human cell lines 293T and hepatic stellate cell lines LX-2 were maintained in Dulbecco’s Modified Eagle Media (DMEM) supplemented with 10% fetal calf serum and 1% penicillin/streptomycin. For assessing αSMA expression, 293T cells were transfected with human CRIPTO expression plasmid (pDNA-CMV-CRIPTO-FLAG), mouse CRIPTO expression plasmid (pCDNA-CRIPTO-WT-FLAG), or pDNA-CMV Empty Vector (control) using Lipofectamine2000 (Life Technologies, Carlsbad, CA, USA), according to the manufacturer’s instructions. For reporter assay, 293T cells were seeded at a density of 50,000 cells in 500 μL of medium in 24-well plates and transfected with 100 ng of αSMA reporter αSMA-luc plasmid, 100 ng of CRIPTO expression vectors or empty vector, and 10 ng of CAGGS-Renilla luciferase. The Firefly luciferase and Renilla luciferase levels in the lysates were measured using Dual Luciferase Assay (Promega, Madison, WI, USA). For overexpression by adenoviral-mediated delivery, 200 multiplicities of infection (MOI) of high titer virus was incubated with the media for 24 h.

### 2.9. Western Immunoblotting

Protein extracts’ liver tissue FFPE sections (2 × 10 μm) were obtained using the QProteome Extraction kit (Qiagen, Hilden, Germany). For protein extracts from cells, they were lysed in cold RIPA lysis buffer (10 mM Tris (pH 8.0), 140 mM NaCl, 1% Triton-X 100, 0.1% C24H39NaO4, 1 mM EDTA (pH 8.0), 0.1% SDS, and 1 mM EGTA plus complete protease inhibitors (Roche, Basel, Switzerland) using a cell scraper. Lysates were passed through a 26-gauge needle. Following a centrifugation step (15 min, 4000 rpm, 4 °C) to remove debris, the protein extract was collected (supernatant). Protein content was quantified using a DC protein assay (Bio-Rad) using serial dilutions of BSA in tissue lysis buffer or Qubit protein high-sensitivity quantification kit. A total of 30 µg were diluted in 4× Laemmli buffer, separated by SDS-PAGE, and transferred to PVDF membranes (0.2 μm, Merck Immobilon™-P PVDF Membrane, ISEQ00005, Merck Millipore AG, Zug, Switzerland). For the protein transfer, the membrane was incubated in 20% ethanol for 10 min and transferred in Tris-Glycine Transfer buffer containing 20% methanol for 1 h, at 400 mA. The following primary antibodies were used in 5% bovine serum albumin diluted in TBS + 0.1% Tween 20 (TBS-T) after blocking in 5% milk-TBS-T: anti-CRIPTO (mouse, 1:1000, 19917, Abcam, Cambridge, UK), anti-CRIPTO (human, 1:1000, kindly provided by Dr. Peter Gray), tubulin (1:1000, MAB1637, Millipore, Burlington, MA, USA), anti-pAKT (S473, 1:1000, 9271S, Cell Signaling, Danvers, MA, USA), anti-pSMAD2 (1:1000 AB3849, Millipore), Anti-FSP1 (1:1000, ab27957, Abcam), Anti-FLAG (1:1000, F7425, Sigma-Aldrich, Buchs, Switzerland), Anti-Collagen type I (1:1000, Southern Biotech, Birmingham, AL, USA), αSMA (1:1000, A2547, Sigma-Aldrich), and Vinculin (1:1000, 3901, clone E1E9V, Cell Signaling). Appropriate secondary HRP antibodies (GE Healthcare, Chicago, IL, USA) were used and detected by chemiluminescence (WesternBright ECL Advasta, K-12045-D50).

### 2.10. Human Plasma CRIPTO Measurements

CRIPTO levels in human plasma samples were measured using ELISA, performed according to the manufacturer’s protocol (R&D systems, Minneapolis, Canada, DY145).

### 2.11. Statistical Analysis

IBM SPSS statistics software (SPSS Inc., Chicago, IL, USA, version 23) was used to perform Spearman tests for correlations. GraphPad Prism software (GraphPad Software, version 5.01, San Diego, CA, USA) was used to perform Student’s *t*-test for the comparison between two groups. *p*-values lower than 0.05 were considered to be statistically significant. The data in the graphs are presented as means ± standard error of the mean (SEM).

## 3. Results

### 3.1. Cripto Expression in Patients with End-Stage Liver Cirrhosis

The presence of liver fibrosis was evaluated by Sirius-red-stained collagen-1α1 deposition and alpha-smooth muscle actin (αSMA, ACTA2)-stained activated stellate cells. Liver tissue of patients with cirrhosis showed significantly higher Sirius-red and αSMA positive signal compared to control tissue, which confirmed the clinical diagnosis of cirrhosis (Figure 1A,B). CRIPTO signal in these tissues was mainly observed in the hepatocytes and was clearly more present in the cirrhotic tissue as compared to control tissue, with 16 of 19 (84.2%) cirrhotic livers showing CRIPTO signal above the highest level in control livers (Figure 1A,B). Furthermore, the results showed a positive correlation between the level of CRIPTO staining and the laboratory MELD scores of the patients (correlation coefficient: 0.577, *p* < 0.003). No significant correlations between CRIPTO and Sirius-red or αSMA staining were observed (data not shown). QPCR analysis also showed elevated *Collagen-1α1*, *αSMA*, and *CRIPTO* mRNA expression levels in cirrhotic liver tissues compared to control liver tissues (Figure 1C). Altogether, these results indicated that livers of patients with cirrhosis express higher levels of CRIPTO compared to control liver tissue and that the level of CRIPTO staining is correlated to the MELD score.

ELISAs were performed to study whether CRIPTO is reflected in the blood of patients with liver cirrhosis. CRIPTO was detected in 31 out of 45 plasma samples of patients with end-stage cirrhosis (69%) and only in two out of the 16 controls (13%) (Chi-square 15.1; *p* < 0.001). The mean CRIPTO level was significantly (*p* = 0.03) higher in the end-stage liver cirrhosis group compared to that of the healthy controls (Table S1). Circulating CRIPTO levels did not correlate with the MELD score (correlation coefficient: 0.151, *p* = 0.310).

One year after LT, CRIPTO levels had significantly decreased in all 31 patients as compared to their initial levels before transplantation (Table S1, Figure 1D,E). A significant decrease in plasma CRIPTO concentration was also observed when the ALD and viral-induced cirrhosis cohorts were analyzed separately (Figure 1D,E). Altogether, these data indicated that CRIPTO levels in plasma decreased significantly once the cirrhotic liver was replaced by a healthy donor liver. Nevertheless, the post-LT plasma CRIPTO levels still remained considerably higher than in the healthy controls, though not statistically significant at the CRIPTO level (*p* = 0.6) but very clear at the positive frequency level (detectable CRIPTO level in patients 27/31 versus 2/16 for controls, Chi-square 24.9; *p* < 0.0001).

### 3.2. CRIPTO Expression in Patients with End-Stage Heart Failure

The presence of cardiac fibrosis was evaluated by the expression of collagen type I, and αSMA-staining was used to visualize the MFBs. Clear collagen type I-positive areas were found in myocardial samples of heart failure patients (Figure S1). CRIPTO expression, however, was minimally detected. Limited expression was observed in the interstitial cells, which did not express αSMA. Additionally, areas with high density of MFBs hardly showed CRIPTO expression (not shown). These observations show that, in contrast to liver fibrosis, CRIPTO was only limitedly expressed in end-stage heart failure patients.

### 3.3. CRIPTO Expression in Mouse Models of Liver Fibrosis

To further study the potential role of CRIPTO in liver fibrosis, we evaluated CRIPTO expression in a CCl_4_-induced mouse model for chronic liver fibrosis (11-week induction; Figure 2A). Liver fibrosis was confirmed by Sirius-red and αSMA staining of the paraffin-embedded liver tissue (Figure 2B). Quantification of the Sirius-red and αSMA staining revealed a higher content of collagen deposition and activated stellate cells in the livers of mice with fibrosis compared to healthy control animals (Figure 2C). Similar to the observations from the human clinical data (Figure 1), CRIPTO staining was pronounced in the liver tissues of mice with fibrosis and mainly observed in the hepatocytes (Figure 2B,C). These findings were further supported by qPCR analysis, which also showed higher *Collagen-1α1*, *αSMA*, and *Cripto* RNA expression in the livers of mice with liver fibrosis compared to the healthy control livers (Figure 2D).

To study the kinetics, dynamics, and stimulus of the reactivated CRIPTO expression, we used an acute liver fibrogenesis model in which mice were subjected to a single shot of CCl_4_ and we subsequently analyzed liver tissues at various time points (Figure 2E). Immunofluorescent co-staining of CRIPTO and αSMA indicated the presence of rare CRIPTO-positive cells in the central vein area, as early as 24 h post CCl_4_ shot (Figure 2F; day 1), followed by a short-term expansion during day 2 when the MFBs (αSMA expressing cells) started to accumulate. The αSMA protein expression levels reached their maximum at day 3 after CCl_4_ administration. Within these first 3 days, only a few CRIPTO-positive cells were observed (Figure 2F; day 3). At the mRNA level, *Cripto* was upregulated at 3 h after CCl_4_ exposure, reached the highest expression on day 1, and rapidly reversed to low levels already on day 3 (Figure 2G). Instead, *αSMA* mRNA levels increased later on (day 2) and reached a maximum level on day 3 (Figure 2H). These results might suggest that CRIPTO expression was silenced in normal liver tissues, was induced as an immediate response to cell injury, and preceded activation of αSMA-positive MFBs. In this model, complete regeneration of the liver and resolution of fibrosis around the damaged portal triads occurred within 7 days from the CCl_4_ administration (Figure 2G,H).

### 3.4. CRIPTO Expression in Mouse Models of Cardiac Fibrosis

**Figure 3:**
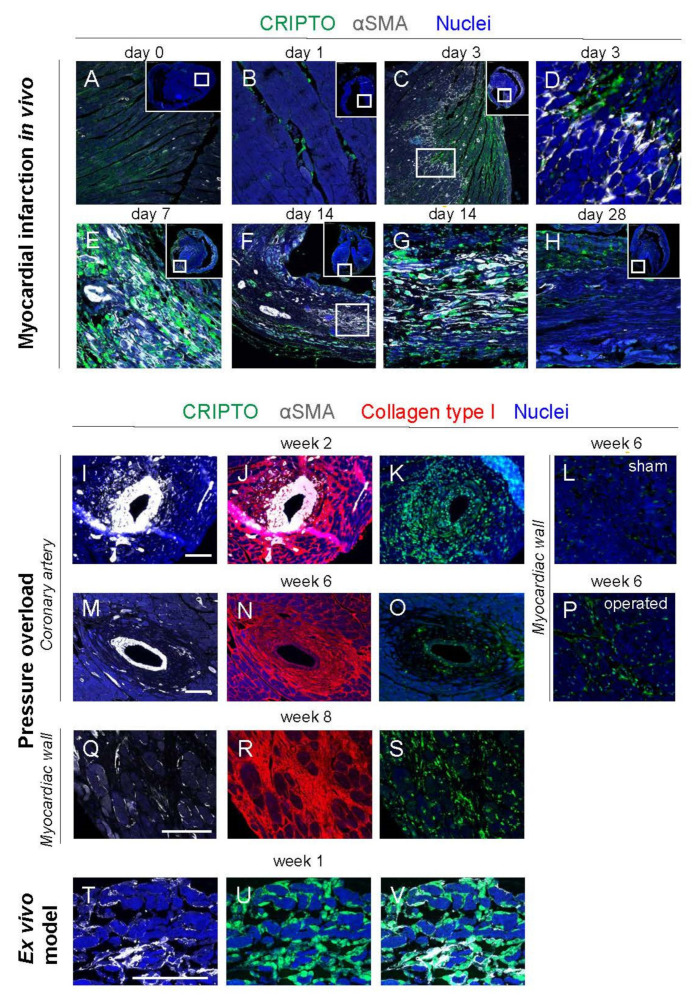
CRIPTO reactivation during wound-healing response to cardiac tissue injury. CRIPTO (green) expression during myocardial infarction associated with αSMA expression (white) and/or collagen type I (red) expression during myocardial infarction ((**A**–**H**); (**A**): day 0 (*n* = 3 independent experiments); (**B**): day 1 (*n* = 3); (**C**,**D**): day 3 (*n* = 3); (**E**): day 7 (*n* = 4); (**F**,**G**): day 14 (*n* = 2); (**H**): day 28 (*n* = 2)), pressure overload ((**I**–**K**): 2 weeks (*n* = 3); (**L**–**P**): 6 weeks (*n* = 4); L=sham (*n* = 1); (**Q**–**S**): 8 weeks (*n* = 1)); around coronary artery (**I**–**O**) and myocardiac wall (**L**,**P**–**S**) and during culture in the ex vivo cardiac fibrosis model ((**T**–**V**): 1 week (*n* = 9)).

To study the potential involvement of CRIPTO in fibrosis further, its expression was also determined in three different mouse models of cardiac fibrosis, i.e., MI, pressure overload, and an ex vivo fibrosis model for whole mouse hearts. CRIPTO was virtually absent in healthy non-operated (Figure 3A) and in sham-operated mouse hearts (Figure 3L). However, upon MI induction by permanent ligation of the left anterior descending artery, a progressive increase in the expression of CRIPTO was observed. At day 1 after MI, single CRIPTO-positive interstitial cells were observed (Figure 3B). On Day 3 after MI, CRIPTO was expressed at the infarction side of the border zone of the infarcted heart, opposite to the αSMA-expressing cells, which were present at the non-infarction side of the border zone (Figure 3C,D). At day 7 after MI, CRIPTO and αSMA were expressed throughout the infarcted area (Figure 3E). At day 14 after MI, the expression of both CRIPTO and αSMA had diminished near the border zone (Figure 3F) and was only present in the center of the infarcted area (Figure 3F,G). At 28 days after MI, both CRIPTO and αSMA had diminished in the infarcted heart (Figure 3H). Pressure overload of the left ventricle was induced by transverse aortic constriction. After 2 weeks, upregulation of CRIPTO and αSMA expression was observed surrounding the coronary arteries (Figure 3I,K), which had decreased 6 weeks after pressure overload, whereas increased collagen type I expression remained (Figure 3J,N), indicating perivascular fibrosis. At 8 weeks after pressure overload, upregulation of CRIPTO, αSMA, and collagen type I were observed in between the cardiomyocytes of the ventricular wall (Figure 3Q–S), indicating interstitial fibrosis. Culturing of whole mouse hearts in the miniature tissue culture system (MTCS) under specific conditions resulted in myocardial fibrosis. Staining for CRIPTO indicated that the formation of αSMA-positive cells coincided with increased CRIPTO staining (Figure 3T–V). Overall, these observations showed a clear association of cardiac fibrosis and temporary CRIPTO expression.

### 3.5. Adenoviral-Mediated in Vivo Overexpression of CRIPTO Leads to Increased Fibrogenesis

**Figure 4:**
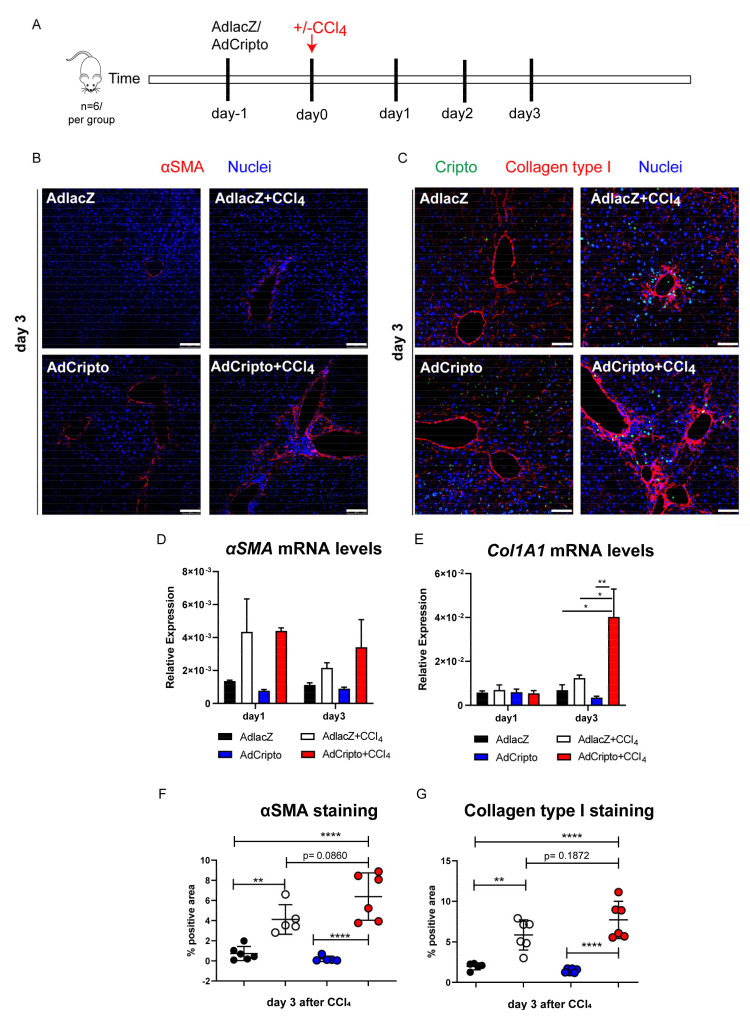
In vivo overexpression of Cripto in combination with CCl_4_ exacerbates fibrosis. (**A**) Experimental setup. Mice received Adenovirus-expressing *Cripto* or *lacZ* (AdlacZ, AdCripto 1 × 10^9^ particles intravenously) 24 h prior to one shot of CCl_4_ (acute liver fibrosis model, 1 mg/kg) and liver tissues were collected at day 1, day 2, day 3, and day 6 (*n* = 6 per treatment group; AdCripto+/−CCl_4_, AdlacZ+/−CCl_4_). (**B**) Representative images of fibrotic liver tissues stained for αSMA (red) on day 3 after CCl_4_. Nuclei were stained with DAPI. Scale bars 100 μm. (**C**) Representative images of fibrotic liver tissues stained for Cripto (green), Collagen type I (red) on day 3 after CCl_4_. Nuclei were stained with DAPI; Scale bars 50 μm. All samples were derived from the same animal experiment. The staining was repeated three independent times. The acquisition and quantification were done on the same experiment for all tissue samples. (**D**,**E**) The mRNA expression levels of *αSMA* and *Col1a1* normalized to *β-actin*. Relative expression ± SEM among *n* = 2 biological replicates per time point and per group. Three technical measurements were obtained per replicate. Ordinary two-way ANOVA, * *p* ≤ 0.05, ** *p* ≤ 0.01. (**F**,**G**) Quantification of αSMA and Cripto staining (average *n* = 3 fields of view per tissue; *n* = 2 mice, mean ± SD). Ordinary two-way ANOVA, ** *p* ≤ 0.01, **** *p* ≤ 0.0001.

To identify whether the observed high CRIPTO levels play a causal role in the induction of wound healing, we combined the acute CCl_4_ model with in vivo overexpression of CRIPTO. Adenovirus-carrying *Cripto*-coding sequence (AdCripto) or control adenoviral particles (AdlacZ) were injected intravenously for optimum uptake by the liver, 24 h prior to the administration of CCl_4_. Liver tissues were analyzed on day 1, day 2 and day 6 after CCl_4_ (Figure 4A). Expression of αSMA and collagen type I deposition was highest at day 3 (Figure 4B,C) after CCl_4_, and was higher in the AdCripto group compared to the control group. CRIPTO-expressing cells were consistently observed after fibrogenic induction by CCl_4_ in both groups (AdlacZ+CCl_4_ and AdCripto+CCl_4_) (Figure 4C). Assessment of the mRNA levels of α*SMA* and *Collagen type I chain* (Col1A1) showed no upregulation after exogenous delivery of *Cripto*-coding sequence (AdCripto group) but only increased following CCl_4_ administration (Figure 4D,E). Interestingly, *Col1A1* mRNA levels were significantly increased in the combined AdCripto+CCl_4_ group on day 3 compared to all other treatment groups (Figure 4E, * *p* < 0.05). Quantification of αSMA staining showed highest levels in the AdCripto+CCl_4_ condition (Figure 4F) (**** *p* < 0.0001 vs. AdlacZ, **** *p* < 0.0001 vs. AdCripto, *p* = 0.08 vs. CCl_4_+AdlacZ). Quantification of Collagen type I staining showed a similar trend, with the highest levels in the CCl_4_+AdCripto condition (Figure 4G) (**** *p* < 0.0001 vs. AdlacZ, **** *p* < 0.0001 vs. AdCripto, *p* = 0.18 vs. CCl_4_+AdlacZ). At the mRNA level, *Cripto* was detectable in liver tissues after AdCripto administration, while it was undetectable in AdlacZ liver tissues (Figure S2A, day 1; groups AdlacZ and AdCripto). The increase of *Cripto* levels due to the CCl_4_ hepatocyte toxicity effect occurred as previously observed after CCl_4_ administration (Figure S2B, day 2, groups CCl_4_+AdlacZ and CCl_4_+AdCripto). At the protein level, we confirmed overexpression of CRIPTO (AdCripto conditions) with the highest levels achieved in combination of CCl_4_+AdCripto (Figure S2C,D). Similarly, both fibrotic markers, Collagen type I and αSMA, were expressed highly upon combined tissue damage and AdCripto administration (Figure S2C,E,F), as assessed in whole tissue protein lysates by western blot analysis.

Using in vitro assays, we showed that CRIPTO is an upstream molecular player involved in the regulation of αSMA expression, a marker of transdifferentiated MFBs, the main cell type responsible for fibrogenesis. Specifically, transient overexpression of human CRIPTO sequence (H-CRIPTO) in 293T cells activated α*SMA-luc* gene reporter (Figure S3A) compared to empty vector control transfected cells (**** *p* ≤ 0.0001). At the protein level, overexpression of either murine or human CRIPTO led to higher levels of phosphorylated AKT and reduced phosphorylated SMAD2 levels (canonical TGFβ) (Figure S3A). Although fibroblast-specific protein-1 (FSP1) and αSMA protein levels were not affected by CRIPTO overexpression, which was probably due to 293T being a non-fibroblast cell line (Figure S3B), at the molecular level CRIPTO induced αSMA gene reporter transcriptional activity (Figure S3A). Similarly, stable overexpression of CRIPTO in LX2 human liver fibroblasts led to *αSMA* gene expression levels (Figure S3C). Overall, CRIPTO upregulation increased the relative level of fibrosis, as shown by more accumulating MFBs and higher expression of fibrotic markers in vivo and in vitro.

### 3.6. Functional Inhibition of CRIPTO by ALK4Fc Ligand Trap Reduces Collagen Deposition and Stimulates Hepatocyte Proliferation

**Figure 5:**
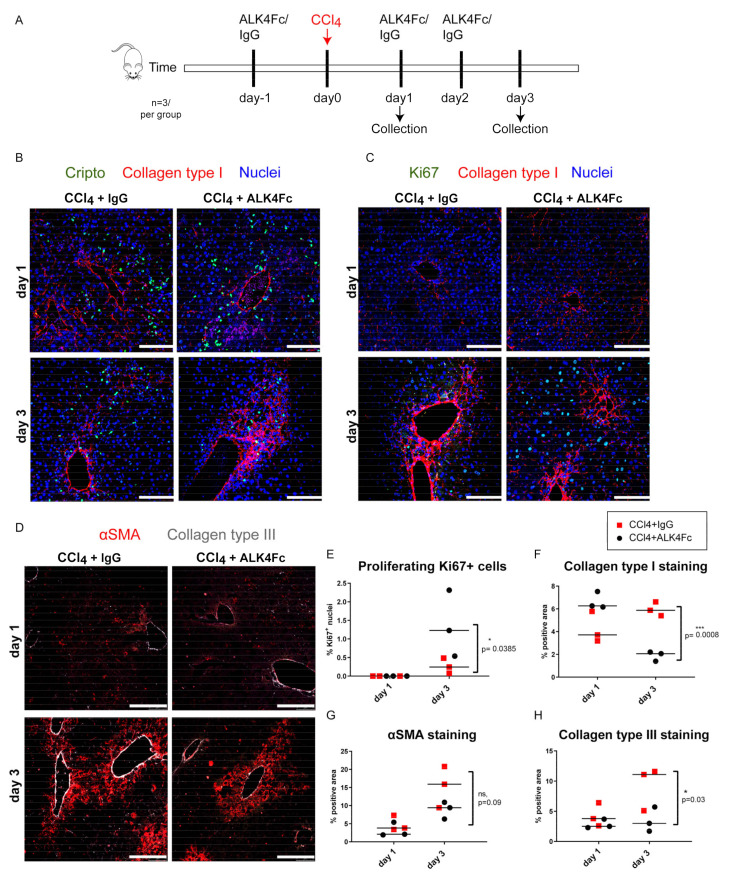
Blocking Cripto function by ALK4Fc leads to reduced collagen deposition in the acute CCl_4_ fibrosis model. (**A**) Experimental setup. Mice received ALK4Fc or IgG vehicle (5 mg/kg) 24 h (day 1) prior to one shot of CCl_4_ (acute liver fibrosis model, 1 mg/kg, day 0) and once daily for 3 days following CCl_4_. Liver tissues were collected at day 1 and day 3 (*n* = 3/ time point and for each of the treatment groups ALK4Fc+CCl_4_ and IgG+CCl_4_). (**B**) Representative images of fibrotic liver tissues stained for Cripto (green) and Collagen type I (red) on days 1 and 3 after CCl_4_. Nuclei stained by DAPI. Scale bars 100 μm. (**C**) Representative images of fibrotic liver tissues stained for Ki67 (green) and Collagen type I (red) on days 1 and 3 after CCl_4_. Nuclei stained by DAPI. Scale bars 100 μm. (**D**) Representative images of fibrotic liver tissues stained for αSMA (red) and Collagen type III (gray) on days 1 and 3 after CCl_4_. Scale bars 100 μm. All samples were derived from the same animal experiment. The stainings in panels (**B**–**D**) were repeated three independent times. (**E**) Quantification of proliferating Ki67+ cells (percentage over the DAPI stained nuclei) (adjusted *p* = 0.0385 *, CCl_4_+ALK4Fc vs. CCl_4_+IgG day 3, two-way ANOVA, median, *n* = 3 biological replicates, each from an average of three fields of view). The acquisition and quantification were done on the same experiment for all tissue samples. (**F**) Quantification of Collagen type I staining (adjusted *p* = 0.0008 ***, CCl_4_+ALK4Fc vs. CCl_4_+IgG day 3, two-way ANOVA, median, *n* = 3 biological replicates, each from an average of three fields of view). (**G**) Quantification of αSMA staining (*p* = 0.09 CCl_4_+ALK4Fc vs. CCl_4_+IgG day 3, two-way ANOVA, median, *n* = 3 biological replicates, each from an average of three fields of view). (**H**) Quantification of Collagen type III staining (adjusted *p* = 0.031 *, CCl_4_+ALK4Fc vs. CCl_4_+IgG day 3; * *p* ≤ 0.05 *** *p* ≤ 0.001, two-way ANOVA, median, *n* = 3 biological replicates, each from an average of three fields of view).

To further understand the dynamics and functional role of CRIPTO in the context of fibrogenesis, we inhibited CRIPTO-secreted protein to sequester CRIPTO-dependent signaling during fibrotic induction in the acute liver CCl_4_ fibrosis model. The ligand trap for the extracellular binding domain of ALK4 receptor, the binding partner of secreted CRIPTO (ALK4Fc) [44,45], was administered i.p. in vivo, 24 h prior to, and at day 1 and day 2 after CCl_4_ exposure (Figure 5A). Liver tissues were analyzed at day 1 and day 3 after CCl_4_ administration for CRIPTO, proliferation (Ki67), and fibrotic (αSMA, Collagen type I and III) markers. CRIPTO-positive cells appeared around the damaged area (indicated by Collagen type I, Figure 5B in red) in both conditions (CCl_4_+IgG, CCl_4_+ALK4Fc) at day 1 and decreased at day 3 (Figure 5B). Proliferating Ki67-positive hepatocytes were apparently outside the fibrotic areas at day 3 (Figure 5C). Furthermore, significantly higher levels of Ki67-positive hepatocytes were observed in the CCl_4_+ALK4Fc liver tissues, compared to the CCl_4_+IgC tissues (Figure 5E, * *p* < 0.05). Collagen type I staining was significantly lower in CCl_4_+ALK4Fc liver tissues (Figure 5C) compared to CCl_4_+IgC (Figure 5F, day3, *** *p* < 0.001). The αSMA expression indicated smaller fibrotic areas around the central veins of the ALK4FC group at day 3 (Figure 5D,G, ns, *p* = 0.09). Similarly, collagen type III signaling was decreased in the CCl_4_+ALK4Fc group (Figure 5D,H, * *p* < 0.05). Overall, the data showed decreased levels of all fibrotic markers and increased levels of regenerating hepatocytes in livers exposed to a combination of CCl_4_ and CRIPTO antagonist (ALK4FC).

## 4. Discussion

Fibrosis may manifest in multiple tissues such as skin, lung, heart, kidney, and liver [46], while antifibrotic treatments directly targeting the process of fibrogenesis are currently not available and activating mechanisms are not fully elucidated [47]. Excessive fibrosis develops on the basis of chronic inflammation and epithelial cell injury due to infections, environmental hazards, or acute tissue damage. Fibrogenesis is a necessary part of the wound-healing response, while fibrosis is aberrant fibrogenesis (extracellular matrix deposition), which does not resolve or facilitate tissue regeneration. Some tissues, such as liver with high regenerative capacity (cell replenishment of the injured cell type), may reverse fibrotic processes if the tissue-damaging stimulus is removed early in the disease development, while other organs, such as the heart, have a low regenerative capacity, resulting in the permanent presence of fibrosis.

In the present study, we assessed whether CRIPTO is expressed during fibrosis in multiple organs in humans and murine models. We observed CRIPTO expression in hepatocytes of human cirrhotic liver tissue, which is in line with the findings in a recent study where we showed that CRIPTO was highly expressed in HCC and was associated with Sorafenib resistance [36]. The levels of CRIPTO expression in the hepatocytes of HCC patients positively correlated with the MELD scoring system for end-stage liver diseases [48,49], illustrating that CRIPTO expression is related to the severity of the disease. In addition, we observed elevated CRIPTO levels in most (69%) of the measured plasma samples of patients with end-stage liver disease, which decreased after the patients underwent LT. This observation is in line with recent findings of Zhang et al., who also observed enhanced CRIPTO levels in the serum of patients with HCV- and HBV-induced cirrhosis [50]. Additionally, in acute and chronic mouse models of liver fibrosis, we observed upregulation of CRIPTO that was indicative of a general and well-preserved role of CRIPTO during fibrogenesis.

Whereas in the MI, pressure overload, and ex vivo mouse models of cardiac fibrosis, CRIPTO was upregulated, only a very limited amount of CRIPTO was observed in the human samples of cardiac fibrosis. The difference can likely be explained by the dynamic expression of CRIPTO during the different stages of fibrosis. The samples were derived from patients that suffered from end-stage heart failure, suggesting the long existence of fibrosis in the heart. In a recent study, CRIPTO was detected in blood samples of patients with planned MI within 1 h [51], suggesting that CRIPTO is expressed shortly after cardiac injury. Together with our observation in the MI model, these data suggest that CRIPTO is mostly present in the early phases of fibrosis.

Although in the majority of plasma samples from patients with end-stage liver cirrhosis we observed elevated CRIPTO levels, in about 30% CRIPTO was undetectable, which is in concordance with a previous study [50]. This might indicate that CRIPTO expression is also dynamic in chronic liver fibrosis.

The CRIPTO-positive cells in the acute CCl_4_ liver model were accumulated in the damaged areas and only partially coinciding with MFBs, indicating that the source of CRIPTO could be a distinct cell type, such as bone marrow-derived fibrocytes, resident progenitors, or hepatic stellate cells prior to their transdifferentiation to MFBs or recruited inflammatory cells [52]. In contrast, in advanced stages (human cirrhosis and chronic mouse liver fibrosis model), CRIPTO is predominantly expressed by epithelial cells (hepatocytes) surrounding but not within the fibrotic areas, indicating that the cellular source of CRIPTO is different in advanced progression and derives from intrinsic cell alterations in damaged hepatocytes. In the injured heart, the expression of CRIPTO is initially, similar to liver, present in interstitial non-MFBs. Shortly thereafter, CRIPTO expression mostly coincides with the expanding population of MFBs until the fibrotic area has been established, after which the CRIPTO expression ceases. However, the CRIPTO expression in the cardiomyocytes is virtually absent. Ex vivo induced cardiac fibrosis similarly led to the appearance of CRIPTO-positive cells, which are, therefore, tissue resident and not derived from a systemic source.

The difference in cell type in which CRIPTO is expressed among organs is intriguing. Whereas in regenerating tissues, such as the liver and skeletal muscle, CRIPTO is expressed in the progenitor cells of the newly formed parenchymal cells, in the non-regenerating heart, CRIPTO remains expressed in the stroma cells. During cardiac development, CRIPTO is required for cardiomyogenesis [53,54,55,56]. The lack of reactivation of CRIPTO in the cardiomyocytes could be hypothesized to preclude myocardial regeneration.

Adenoviral CRIPTO overexpression in the acute CCl_4_ liver model resulted in higher levels of fibrotic markers, whereas inhibition of CRIPTO by the ALK4Fc ligand trap [45] led to improved hepatocyte proliferation and significantly reduced fibrosis. These observations indicate that CRIPTO is not simply a mere consequence of the tissue damage or fibrosis but that it may regulate the cascade of fibrosis by inducing myofibroblast formation and collagen production. The expression of CRIPTO in the heart shortly after MI concomitant with, and even preceding, the appearance of MFBs suggests a similar role for CRIPTO in cardiac fibrosis.

From a molecular point of view, the mechanism causing the upregulation of CRIPTO and the enhanced fibrotic response remains to be further addressed. NANOG, a regulator of CRIPTO expression, is expressed in hepatocytes during fibrogenesis [36,57] and expressed in cardiac interstitial cells [58], which could, thus, contribute to the CRIPTO expression during fibrogenesis [59]. Furthermore, CRIPTO expression has been shown to be involved in the activation of the well-known SMAD pathways, which could promote fibrogenesis and eventually HCC formation [33,57,60] and regulate the cardiac fibrotic response [61]. It has been shown that inducing cell damage to HepG2 cells leads to the upregulation of CRIPTO, which initiates apoptotic resistance and increased proliferation via NF-κB/Survivin pathways [62]. A similar mode of CRIPTO reactivation may occur in vivo, in which CRIPTO may become re-expressed as a response to cellular injury in order to promote tissue regeneration by orchestrating the reactivation of both fibrogenic cells and of quiescent hepatocytes.

CRIPTO has a profibrotic role in both heart and liver fibrosis, and we showed for the first time that CRIPTO reactivation is linked to tissue homeostasis, wound-healing response, and fibrosis and is conserved in different tissues with low and high regenerative capacity.

Altogether, the observations from this study warrant further research to disentangle whether CRIPTO has a functionally relevant role in fibrotic diseases, with biomarker and therapeutic value, while targeting its activation can alleviate the extent and progression of fibrosis.

## Supplementary Materials

The following are available online at https://www.mdpi.com/article/10.3390/cells10123325/s1, Figure S1: Cripto expression in patients with end-stage heart failure, Figure S2: Cripto overexpression in liver tissues increases protein levels of fibrotic markers, Figure S3: CRIPTO is an upstream regulator of αSMA expression, Table S1: Patient characteristics, Table S2: Primer sequences.
