# Supplementary material for: Oncofetal Protein CRIPTO Is Involved in Wound Healing and Fibrogenesis in the Regenerating Liver and Is Associated with the Initial Stages of Cardiac Fibrosis
Source: Cells. 2021 Nov 26;10(12):3325. doi: 10.3390/cells10123325 (PMC8699799; doi:10.3390/cells10123325)
Supplement: Supplementary file 1 [file cells-10-03325-s001.zip › cells-1463313-supplementary.pdf]

cells

MDPI

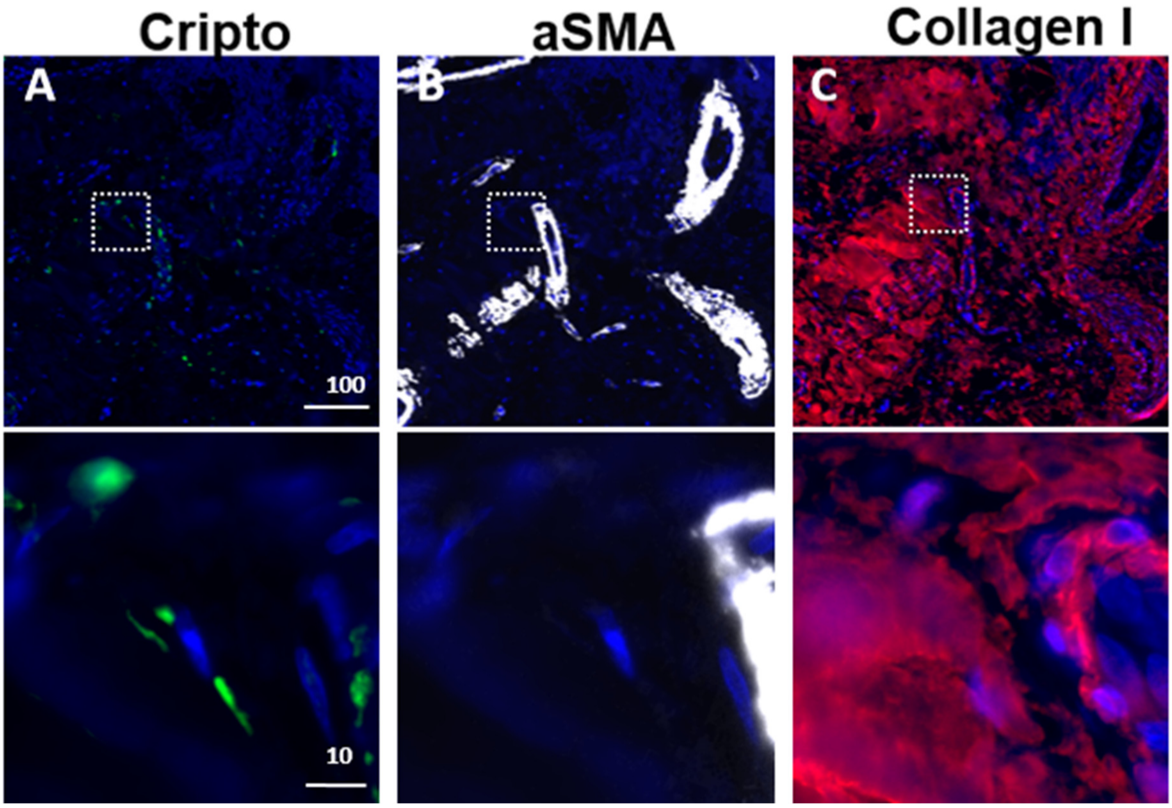


**Figure S1. Cripto expression in patients with end-stage heart failure.** Cardiac tissue samples of patients with end-stage heart failure (n=5) were stained for CRIPTO (A) αSMA (B) and collagen type I (C). Scale bar 100 µm.

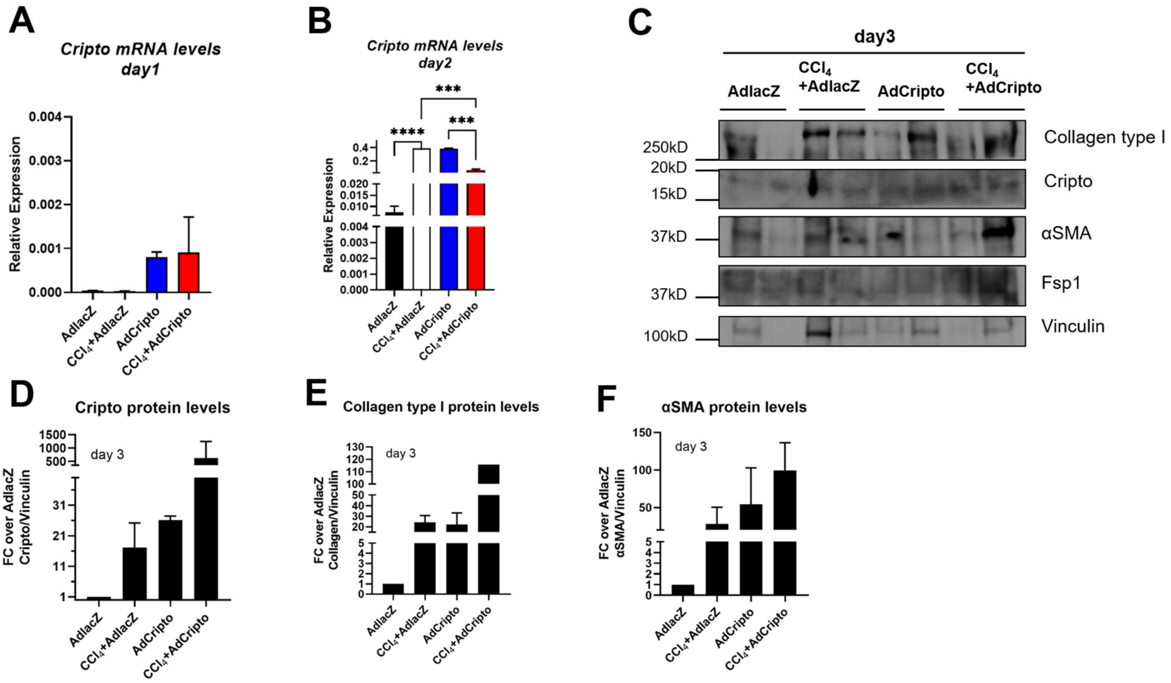


**Figure S2. Cripto overexpression in liver tissues increases protein levels of fibrotic markers**. (A-B) mRNA expression levels of *Cripto* normalized to *β-actin* in the AdCripto+/-$CCl_4$, AdlacZ+/-$CCl_4$ liver tissues at day 1 (A) and day 2 post $CCl_4$ administration. (B) Relative expression ±SEM among n=2 biological replicates per time point and per group. Three technical measurements were obtained per replicate. Ordinary two-way ANOVA, ***p≤0.001, ****p≤0.0001. (C) Protein levels of Collagen type I, CRIPTO, *α*SMA and Fibroblast-specific protein-1 (FSP1) measured by Western blotting in liver tissue FFPE extracts of the AdCripto+/-$CCl_4$ and AdlacZ+/-$CCl_4$ groups. N=2 biological replicates per group. Time point: day 3 post toxin administration. Vinculin was used as protein loading control. Representative of two independent experiments. (D-F) Quantification of protein levels of CRIPTO (D), COLLAGEN Type I (E) and *α*SMA (F) based on the Western blotting of panel (C). Data were obtained after normalization to the mean intensity of the n=2 biological replicates of the control group (AdlacZ), and to the intensity of the protein loading control (vinculin) for every sample. Mean fold change over AdlacZ ±SEM (n=2).

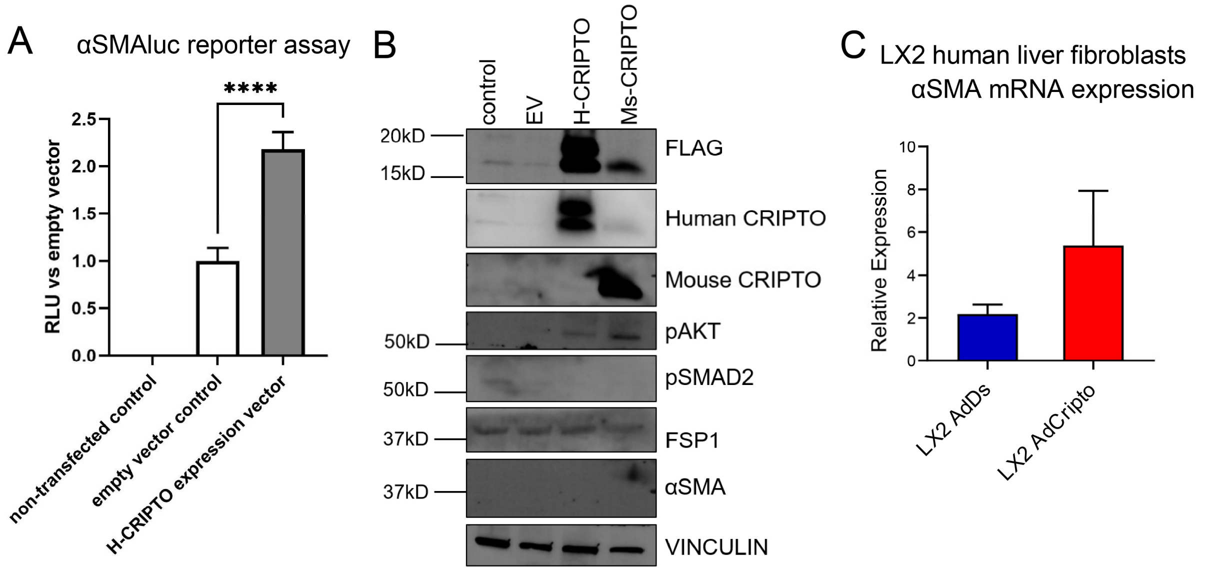


**Figure S3. CRIPTO is an upstream regulator of $\alpha$SMA expression.** (A) *In vitro* $\alpha$SMAluc reporter assay on 293T cells, transfected with empty vector or full length human CRIPTO-expressing plasmid (H-CRIPTO). Relative Luciferase (RLU) measurements were obtained 48 hours after transfection and normalized to the empty vector control. Mean±SD from quadruplicate technical replicate measurements. Unpaired t-test. ****$p \leq 0.0001$. (B) Protein levels measured by Western blotting in 293T cells transfected with empty vector, mouse CRIPTO-FLAG (Ms-CRIPTO) plasmid, human CRIPTO-FLAG (H-CRIPTO) plasmid. Proteins detected: human/mouse CRIPTO, FLAG (to detect CRIPTO-FLAG protein of the mouse and human expression vectors), phosphorylated AKT (pAKT), phosphorylated SMAD2 (pSMAD2), Fibroblast-specific protein-1 (FSP1) and $\alpha$SMA. Time point: 48 hours after transfection. Vinculin was used as protein loading control. Representative of two independent experiments. (C) *$\alpha$SMA* mRNA expression in LX2 human liver fibroblast cell line after adenoviral-mediated CRIPTO (AdCRIPTO) overexpression *in vitro*. AdDs; control adenovirus containing dsRED fluorescent protein.

**Table S1. Patient characteristics**. Data presented as median (range) for continuous variables and percentage (number) for categorized variables.

| Variable | Healthy controls (N=16) | Pre-LT (N=45) | Post-LT (N=45) |
|---|---|---|---|
| Gender (male), % (n) | | | 78% (35) |
| Age (median, range) | 50% (8) | | 54 (42-69) |
| | 29 (23-65) | | |
| Aetiology | | | |
| - Alcoholic liver disease | | | 25 |
| - Viral Hepatitis | | | 20 |
| | | | |
| Blood (median, range) | | | |
| - AST (U/L) | | 72 (24-517) | 27 (11-240) |
| - ALT (U/L) | | 37 (15-360) | 25 (8-401) |
| - INR | | 1.2 (1-2.4) | 1.0 (0.9-2.4) |
| - ALP (U/L) | | 130 (50-555) | 88 (47-487) |
| - Creatinin (μmol/L) | | 92 (34-171) | 111 (68-204) |
| - γGT (U/L) | | 43 (7-374) | 39 (9-1395) |
| - Sodium (mmol/L) | | 138 (124-156) | 142 (134-148) |
| - Bilirubin (μmol/L) | | 46 (5-593) | 12 (5-29) |
| - Platelet count (10^9/L) | | 72 (30-142) | 144 (93-243) |
| Cripto plasma (pg/ml) | | 1381 (0-12108) | 357 (0-5314) |
| Clinical scores | 0 (0-818) | | |
| - MELD | | 15 (8-33) | 10 (6-18) |

LT = Liver Transplantation, ALD = Alcoholic Liver Disease, AST = Aspartate aminotransferase, ALT = Alanine aminotransferase, INR = International Normalized Ratio, ALP = Alkaline phosphatase

**Table S2. Primer sequences.**

| Gene | Forward | Reversed |
|---|---|---|
| **Human** | | |
| $\alpha$-smooth muscle actin | TTGCCTGATGGGCAAGTGAT | GTGGTTTCATGGATGCCAGC |
| Collagen-1$\alpha$1 | GGAACTTGGGGCAAGACAGT | GAGGGAACCAGATTGGGGTG |
| Cripto (human) | CACGATGTGCGCAAAGAGAA | TGACCGTGCCAGCATTTACA |
| β-actin (human) | AATGTCGCGGAGGACTTTGATTGC | GGATGGCAAGGGACTTCCTGTAAA |
| **Mouse** | | |
| $\alpha$-smooth muscle actin | GTCCCAGACATCAGGGAGTAA | TCGGATACTTCAGCGTCAGGA |
| Collagen-1$\alpha$1 | GTGGAAACCCGAGCCCTGCC | TCCCTTGGGTCCCTCGACGC |
| Cripto (mouse) | CGCCAGCTAGCATAAAAGTG | CCCAAGAAGTGTTCCCTGTG |
| β-actin (mouse) | GGGGTGTTGAAGGTCTCAAA | AGAAAATCTGGCACCCC |
